# uPAR-directed immunotherapies in solid tumors: current advances and future perspectives

**DOI:** 10.3389/fphar.2026.1961182

**Published:** 2026-09-21

**Authors:** Filomena Napolitano, Nunzia Montuori

**Affiliations:** 1 Department of Translational Medical Sciences, University of Naples Federico II, Naples, Italy; 2 Center for Basic and Clinical Immunology Research (CISI), WAO Center of Excellence, University of Naples Federico II, Naples, Italy

**Keywords:** CAR T cell therapy, immunotherapy, solid tumors, uPAR, urokinase plasminogen activator receptor

## Abstract

In cancer, elevated expression of the urokinase-type plasminogen activator receptor (uPAR) is a well-established adverse prognostic factor associated with enhanced tumor invasion, metastatic dissemination, cellular proliferation, and resistance to conventional therapies. Genetic ablation or silencing of uPAR has been shown to impair multiple protumorigenic functions both *in vitro* and *in vivo*, underscoring its pivotal role in tumor initiation, progression, and metastatic evolution. These findings establish uPAR not only as a clinically relevant prognostic biomarker but also as an attractive therapeutic target. Beyond its expression on malignant cells, uPAR is abundantly expressed within the tumor microenvironment (TME), including stromal components and immunosuppressive cell populations that actively support tumor progression and immune evasion. Notably, uPAR is highly expressed in several solid tumors characterized by *TP53* and *RAS* pathway alterations. These tumors exhibit a progenitor-like phenotype sustained by a senescence-associated, uPAR-positive stromal niche, further highlighting the contribution of uPAR to tumor plasticity and therapeutic resistance. Collectively, these observations provide a strong biological rationale for the development of uPAR-directed immunotherapeutic strategies aimed at simultaneously targeting tumor cells and the supportive TME. Such approaches may be particularly effective in aggressive and treatment-refractory malignancies, including glioblastoma, non-small cell lung cancer, and ovarian cancer. This review summarizes the current understanding of the biological role of uPAR in solid tumors and discusses the rationale for its therapeutic targeting. Furthermore, it provides a comprehensive overview of the principal uPAR-directed immunotherapeutic strategies currently under investigation, including chimeric antigen receptor (CAR) T-cell therapies, multi-epitope vaccines, monoclonal antibodies, and radioligand-based approaches. Collectively, these emerging modalities position uPAR as a versatile and broadly applicable immunotherapeutic target with the potential to address several of the major challenges associated with the treatment of solid tumors.

## 1 Background

Traditionally, cancer treatment has relied on surgery, radiotherapy, and chemotherapy. Although these modalities remain the cornerstone of clinical management for many malignancies, their therapeutic efficacy is frequently limited by metastatic disease, incomplete tumor eradication, treatment resistance, and substantial toxicity to healthy tissues (Zafar et al., 2025). The advent of cancer immunotherapy has revolutionized the therapeutic landscape by shifting the focus from targeting malignant cells to modulating the complex interactions within the tumor microenvironment (TME), a dynamic ecosystem comprising immune cells, stromal cells, blood vessels, fibroblasts, and the extracellular matrix (ECM) (Colley et al., 2024; Li et al., 2023). Current immunotherapeutic strategies include immune checkpoint inhibitors, monoclonal antibodies, therapeutic cancer vaccines, and adoptive cell therapies, particularly chimeric antigen receptor (CAR) T-cell therapy. While these approaches have produced unprecedented and durable clinical responses in selected malignancies, their broader clinical application remains limited by several challenges, including low response rates in many solid tumors, primary and acquired resistance, antigen heterogeneity, an immunosuppressive TME, and immune-related adverse events. Consequently, the identification and validation of novel immunotherapeutic targets capable of simultaneously enhancing anti-tumor immunity and overcoming the immunosuppressive barriers of the TME have become major priorities in cancer research, with the goal of improving the efficacy and durability of treatment responses (Zhang L. et al., 2026).

In this context, the urokinase-type plasminogen activator receptor (uPAR) has emerged as a highly promising immunotherapeutic target owing to its multifaceted role in tumor progression, ECM remodeling, and regulation of TME. Beyond its established prognostic significance, uPAR contributes to multiple hallmarks of cancer by orchestrating pericellular proteolysis, promoting tumor cell invasion and metastasis, and modulating signaling pathways involved in cell proliferation, survival, and immune evasion (Li et al., 2021).

Classically, uPAR is a central component of the plasminogen activation system, also referred to as the fibrinolytic system, where it binds its cognate ligand, urokinase-type plasminogen activator (uPA), thereby localizing and enhancing the conversion of plasminogen into plasmin. This proteolytic cascade mediates ECM degradation and activates multiple proteases and growth factors, thereby facilitating tissue remodeling, tumor invasion, angiogenesis, and metastatic dissemination (Napolitano and Montuori, 2021; Napolitano and Montuori, 2022; Napolitano et al., 2023).

Human uPAR is encoded by the *PLAUR* gene, located on chromosome 19q13.31. The canonical *PLAUR* gene comprises seven exons encoding a 335-amino acid precursor that undergoes extensive post-translational processing, including N-linked glycosylation and glycosylphosphatidylinositol (GPI)-anchorattachment, yielding the mature receptor expressed on the cell surface (Roldan et al., 1990).

Structurally, uPAR is a 55–60 kDa GPI-anchored cell surface glycoprotein composed of three homologous Ly6/uPAR (LU) domains (DI, DII, and DIII) connected by flexible linker regions that confer considerable conformational plasticity and enable dynamic interactions with multiple binding partners. This structural organization underlies the receptor’s ability to coordinate both proteolytic and non-proteolytic signaling pathways regulating cell adhesion, migration, invasion, tissue remodeling, and intracellular signal transduction (Smith and Marshall, 2010).

In addition to its membrane-bound form, uPAR can be released from the cell surface through phospholipase-mediated cleavage or proteolytic shedding of its GPI anchor, generating soluble uPAR (suPAR), a circulating molecule that is detectable in plasma, serum, urine, cerebrospinal fluid, and other biological fluids (Xu et al., 2024; Napolitano et al., 2021; Burcsár et al., 2021; Tzanakaki et al., 2012; Gustafsson et al., 2011; Klim et al., 2024). Rather than representing an inactive by-product of receptor turnover, suPAR retains substantial biological activity and mirrors the level of immune activation and tissue remodeling (Rasmussen et al., 2021). Elevated suPAR concentrations have been reported in a broad spectrum of pathological conditions, including chronic inflammatory disorders, infectious diseases, cardiovascular diseases, kidney diseases, and numerous solid and haematological malignancies (Napolitano et al., 2021; Napolitano et al., 2025; Belvederi et al., 2025), supporting its role as a systemic biomarker of chronic inflammation and disease severity (Manfredi et al., 2023).

In cancer, increased circulating suPAR levels correlate with tumor burden, advanced disease stage, metastatic dissemination, and poor clinical outcome in multiple tumor types, including colorectal, pancreatic, gastric, ovarian, breast, and prostate cancers. Accordingly, suPAR has emerged as a promising minimally invasive biomarker for patient stratification, prognostic assessment, treatment monitoring, and early detection of disease recurrence (Liu et al., 2017a).

Both membrane-bound uPAR and suPAR can undergo proteolytic cleavage within the linker region connecting domains DI and DII, generating truncated forms that lack the DI domain and consequently display reduced affinity for uPA, together with distinct signaling properties (Ploug and Ellis, 1994). Importantly, proteolytic processing exposes the highly conserved chemotactic sequence ^88^SRSRY^92^ within the DI–DII linker region. This motif interacts with members of the formyl peptide receptor (FPR) family, a group of G protein-coupled chemoattractant receptors that play central roles in innate immunity, host defense, and the regulation of inflammatory responses (Rossi et al., 2016; Rossi et al., 2015; Napolitano et al., 2024). Thus, proteolytic cleavage not only regulates uPA-dependent proteolysis but also expands the repertoire of uPAR-mediated signaling through FPR activation. In addition, multiple uPAR isoforms generated through alternative splicing have been described, further increasing the structural and functional heterogeneity of the receptor (Stewart and Sayers, 2009).

uPAR is expressed by a variety of cell types, including immune cells, endothelial cells, smooth muscle cells, kidney podocytes, fibroblasts, and keratinocytes (Ismail et al., 2021; Napolitano et al., 2018). Underphysiological conditions, uPAR expression is generally low and restricted to processes requiring controlled tissue remodeling, such as wound healing, immune cell trafficking, and inflammation. However, in malignant tissues, uPAR is overexpressed not only by tumor cells but also by stromal and immune components of the TME, including cancer-associated fibroblasts, macrophages, neutrophils, and endothelial cells (van Dam et al., 2021).

Beyond its involvement in proteolytic cascade, uPAR also acts as a signaling platform by interacting with integrins, receptor tyrosine kinases, and G-protein coupled receptors, thereby activating intracellular pathways involved in proliferation, survival, epithelial-mesenchymal transition (EMT), migration, and resistance to therapy (Zhai et al., 2022).

Several studies have demonstrated that uPAR exerts pro-survival activity in diverse cancer cells through activation of ERK1/2 and associated anti-apoptotic signaling pathways (Smith and Marshall, 2010). In pancreatic ductal adenocarcinoma (PDAC), uPAR has recently been identified as a major effector of *TP53* mutations in gemcitabine-treated cells (Zampieri et al., 2025). Specifically, mutant p53 promotes the upregulation of *PLAUR*, the gene encoding uPAR, following gemcitabine exposure, resulting in increased uPAR-dependent activation of the ERK1/2 signaling axis and enhanced cell survival, migration, and invasion. Notably, silencing *TP53* markedly reduced gemcitabine-induced *PLAUR* expression and ERK1/2 activation, supporting a functional link between mutant p53 signaling and uPAR expression. A regulatory connection between oncogenic RAS signaling and uPAR expression has also been described. Activated K-Ras promotes *PLAUR* transcription through AP-1-dependent mechanisms involving c-Jun, JunD, c-Fos, and Fra-1 binding to AP-1 elements within the uPAR promoter (Okan et al., 2001). Consistent with this mechanism, silencing *KRAS* was shown to reduce uPAR levels in PDAC cells, whereas mutant KRAS promoted uPAR-associated MEK/ERK signaling and a mesenchymal phenotype (Peng et al., 2023). Similarly, *RAS* mutational status has been functionally associated with uPAR overexpression in non-small cell lung cancer (NSCLC) and colorectal cancer (CRC) cell lines (Mauro et al., 2017). Collectively, these findings suggest that oncogenic TP53 and RAS signaling may converge on uPAR regulation, thereby contributing to downstream pathways involved in tumor cell survival, migration, invasion, and therapeutic resistance.

Importantly, the different uPAR isoforms described above may exert distinct biological functions in tumor invasion and immune regulation. Full-length, GPI-anchored uPAR serves as a primary docking site for uPA on both cancer cells and surrounding stromal cells. Upon binding to uPAR, pro-uPA is converted into its active form, uPA, which subsequently cleaves plasminogen to generate active plasmin. Plasmin can then cleave and activate downstream matrix metalloproteinases (MMPs), thereby amplifying pericellular proteolysis. The resulting activation of fibrinolytic proteases and MMPs promotes ECM degradation and the release of bioactive growth factors, including epidermal growth factor (EGF), thereby facilitating tumor cell invasion and metastatic dissemination (Danø et al., 2005). In this context, the significant upregulation of uPA in Stroma AReactive Invasion Front Areas (SARIFA)-positive colorectal and gastric cancer tumor cells, together with increased expression of uPAR in adjacent stromal cells, suggests a potential tumor-stroma receptor-ligand interaction that may promote pericellular proteolysis and ECM remodeling at the tumor invasion front, potentially contributing to tumor invasion into the surrounding adipose tissue (Reitsam et al., 2026). Truncated membrane-bound uPAR (II–III), by exposing SRSRY sequence, can interact with FPRs and promote chemotactic signaling and cell migration. Thus, truncated uPAR (II–III) may regulate tumor-cell motility through mechanisms that are distinct from the uPA-dependent pericellular proteolysis mediated by full-length uPAR (Magnussen et al., 2017). Consistent with this concept, inhibition of uPAR cleavage reduced cell migration in oral squamous cell carcinoma (OSCC) cells, whereas inhibition of uPA activity reduced invasion, indicating that uPAR processing and uPA-dependent proteolysis can regulate distinct components of the invasive phenotype (Magnussen et al., 2017).

Soluble uPAR, which comprises different molecular species, including full-length suPAR and cleaved suPAR (II–III), may also exert distinct biological effects. Whereas full-length suPAR retains the ability to bind uPA and other extracellular ligands, cleaved suPAR (II–III) can expose the SRSRY sequence and act as a soluble chemotactic mediator. In cancer cells, conditioned medium containing either full-length suPAR or cleaved suPAR (II–III) stimulated migration of cells expressing low endogenous levels of uPAR, supporting a paracrine role for soluble uPAR species in tumor-cell motility (Magnussen et al., 2017). Given its crucial role in cancer initiation and progression, uPAR has emerged as a potential “Trojan horse” for targeted cancer therapy (Metrangolo et al., 2021). uPAR-targeted therapy has gained increasing attention and has shown promising results in preclinical studies. Several approaches, mainly immunotherapeutic strategies, have been explored and are currently under investigation. Their rationale, as well as their potential advantages and limitations, will be discussed in the following sections.

## 2 Rationale for targeting uPAR in cancer immunotherapy

The identification of suitable tumor-associated antigens remains a major challenge in the development of effective cancer immunotherapies. An ideal therapeutic target should exhibit high and preferential expression in tumor tissues while displaying minimal expression in normal tissues, thereby maximizing antitumor efficacy and minimizing on-target, off-tumor toxicity. In this context, accumulating preclinical and clinical evidence provides a strong biological rationale for targeting uPAR in cancer. Its widespread overexpression across multiple solid malignancies, restricted expression in most healthy adult tissues, and multifaceted role in tumor progression, metastasis, and immunosuppression make uPAR a particularly attractive candidate for next-generation immunotherapeutic strategies.

First, uPAR is overexpressed in a wide range of aggressive solid malignancies, including breast cancer (Indira Chandran et al., 2015), pancreatic cancer (Aronen et al., 2021), colorectal cancer (Liu et al., 2017b), ovarian cancer (Bifulco et al., 2014), non-small-cell lung cancer (Cobos et al., 2003), and brain cancer (Maich et al., 2026), where its expression often correlates with poor prognosis, metastatic potential, and resistance to therapy.

Second, uPAR expression is not restricted to malignant cells but is also enriched in multiple stromal and immunosuppressive components of TME, including cancer-associated fibroblasts (CAFs), tumor-associated macrophages (TAMs), endothelial cells, and tumor-infiltrating lymphocytes (TILs) (Metrangolo et al., 2021). In multiple myeloma, uPAR contributes to the activated phenotype and functional properties of CAFs during disease progression, promoting their proliferative and invasive capacities. These effects are markedly attenuated by selective uPAR silencing, highlighting the role of uPAR in regulating CAF-mediated tumor-supportive functions (Ciavarella et al., 2017). Additionally, uPAR-expressing melanoma-derived exosomes promote angiogenesis by enhancing VE-cadherin and EGFR expression and activating ERK1/2 signaling in endothelial cells, providing further evidence that uPAR contributes to tumor progression not only through direct effects on cancer and stromal cells but also through extracellular vesicle-mediated communication within the TME (Biagioni et al., 2021). This widespread distribution highlights uPAR as a central regulator of the dynamic crosstalk between tumor cells and the surrounding stroma, thereby contributing to several hallmarks of cancer progression (Smith and Marshall, 2010).

Beyond its expression in stromal cells, uPAR also plays a pivotal role in shaping the immune landscape of TME. Supporting this concept, high infiltration of M2-polarized TAMs is positively associated with increased uPAR expression in breast cancer, suggesting a close interplay between macrophages and the uPA/uPAR axis that contributes to a more aggressive tumor phenotype (Lindsten et al., 2017). Consistent with this observation, uPAR expression in several malignancies, including breast cancer, pancreatic cancer, and glioblastoma enhances the ability of tumor cells to induce M2 macrophage polarization, thereby reinforcing an immunosuppressive and tumor-promoting niche (Hu et al., 2014).

The immunomodulatory role of uPAR extends beyond macrophage polarization. In clear cell renal cell carcinoma, elevated PLAUR expression is associated with a distinct immune infiltrate, positively correlating with plasma cells, T cells, M0 macrophages, and neutrophils, while negatively correlating with resting memory CD4^+^ T cells, natural killer (NK) cells, and resting mast cells. Notably, PLAUR expression also shows a strong positive correlation with multiple immune checkpoint genes, suggesting that uPAR contributes to the establishment of an immunosuppressive TME (Wang et al., 2022). Similarly, in glioma, high PLAUR expression is associated with reduced CD8^+^ T-cell infiltration, increased M2 macrophage accumulation, and elevated expression of immune checkpoint molecules and immunosuppressive cytokines, highlighting uPAR as a key regulator of immune evasion and a promising target for immunotherapeutic intervention (Zeng et al., 2021).

Emerging evidence further implicates uPAR-positive extracellular vesicles (EVs) in systemic immune regulation. In patients with metastatic melanoma, elevated levels of uPAR^+^ plasma-derived EVs are associated with poor clinical responses to immune checkpoint blockade (Porcelli et al., 2021). The authors propose that EVs released by tumor cells, dendritic cells (DCs), and CD8^+^ T cells may contribute to the establishment of a systemic immunosuppressive environment that limits effective antitumor immunity. In agreement with these findings, Laurenzana et al. suggest that uPAR^+^ EVs identify highly glycolytic tumors capable of creating a metabolically immunosuppressive microenvironment (L et al., 2017).

Collectively, these findings indicate that uPAR functions not only as a regulator of tumor invasion and metastasis but also as a key orchestrator of stromal remodeling and immune suppression. Therefore, uPAR-directed therapies offer the unique opportunity to simultaneously target malignant cells and tumor-supportive stromal and immune compartments, providing a multifaceted strategy to enhance anticancer efficacy.

In addition to these characteristics, the restricted expression of uPAR in normal tissues, except for subsets of myeloid cells, reduces the likelihood of severe systemic toxicity and supports the development of selective therapeutic strategies with a potentially favorable safety profile (Metrangolo et al., 2021; Eefsen et al., 2025).

Another important characteristic of uPAR is its accessibility on the cell surface. As a GPI-anchored receptor exposed at the plasma membrane, uPAR is highly amenable to antibody-based targeting, immune-cell redirection strategies, and ligand-mediated delivery systems. Indeed, GPI-anchored proteins have attracted increasing interest in cancer research because of their involvement in tumor progression, immune evasion, and modulation of cell signaling pathways, highlighting their potential utility as diagnostic biomarkers and targets for precision immunotherapy (Hussein et al., 2020).

Another important finding is that uPAR is highly expressed in senescent-like cells associated with fibrosis and therapy-induced senescence, and preclinical studies suggest that targeting uPAR may selectively eliminate these cells, thereby reducing fibrosis and improving tissue function (Eskiocak et al., 2026; Ming et al., 2025; Amor et al., 2024).

Taken together, these evidence suggest that therapeutic targeting of uPAR may exert dual anti-tumor effects by directly eliminating uPAR-expressing cells while simultaneously disrupting pro-tumorigenic signaling and alleviating the immunosuppressive TME.

## 3 uPAR-directed immunotherapeutic strategies

The biological features of uPAR described above provide a strong rationale for its exploitation as a therapeutic target in cancer immunotherapy. These properties have driven the development of diverse uPAR-directed immunotherapeutic strategies, such as antibody-based approaches, targeted drug delivery systems, and advanced cell-based therapies. In this section, we review the current landscape of uPAR-targeted immunotherapies, focusing on their mechanisms of action, preclinical and clinical development, therapeutic potential, and the key challenges. Finally, we propose an integrative translational framework that links uPAR biology with therapeutic targeting and clinical implementation, providing a conceptual basis for the future development of uPAR-based immunotherapies (Figure 1).

**FIGURE 1:**
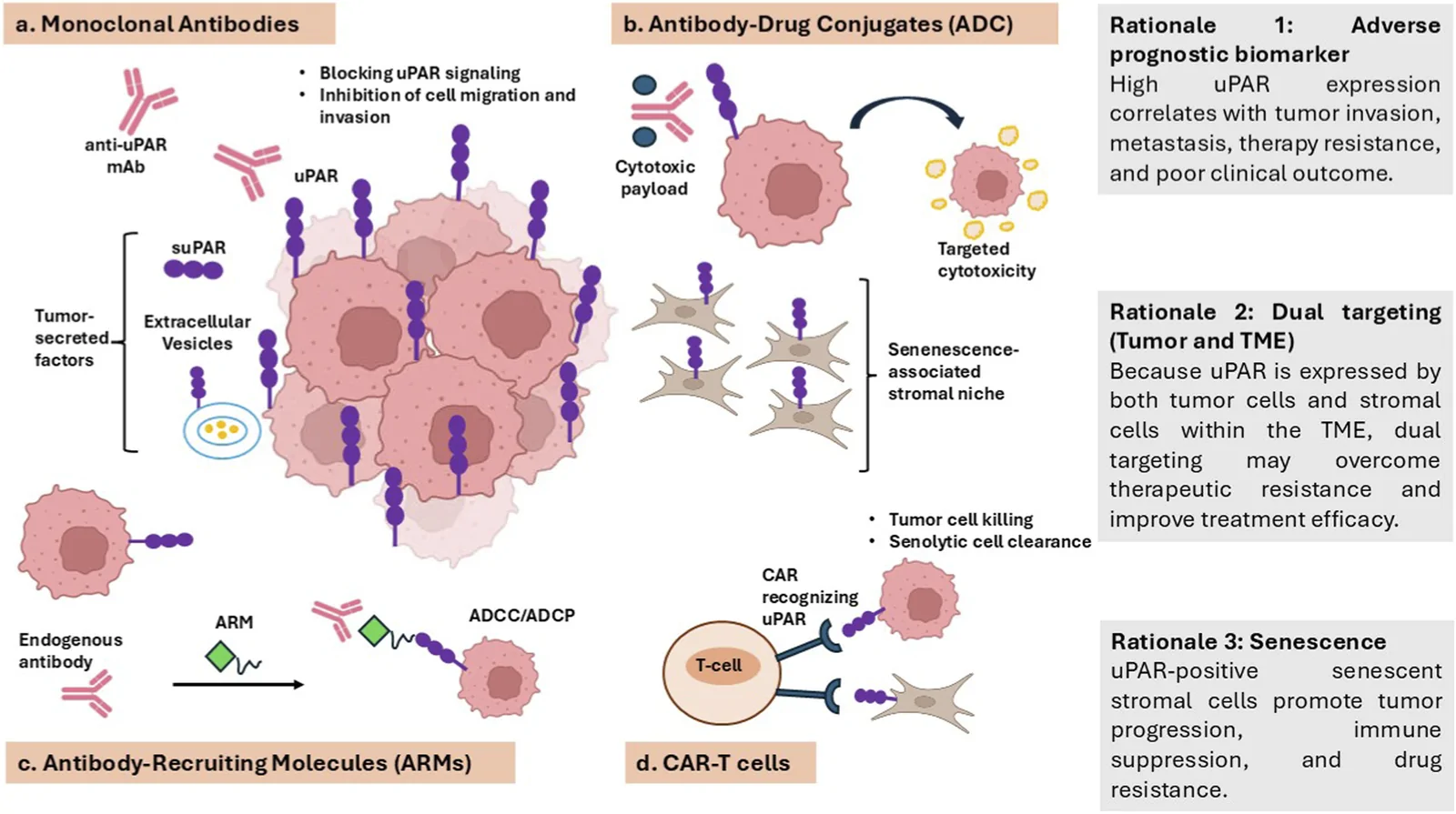
Schematic overview of uPAR-directed immunotherapeutic strategies in cancer. uPAR is overexpressed by tumor cells and multiple stromal cell populations within the tumor microenvironment (TME), where it promotes tumor progression, invasion, metastasis, therapy resistance, and immune suppression. In addition to its membrane-bound form, uPAR is released as soluble uPAR (suPAR) and can also be associated with extracellular vesicles (EVs), contributing to the establishment of an immunosuppressive TME. These features make uPAR an attractive therapeutic target for diverse immunotherapeutic strategies. **(a)** Monoclonal antibodies (mAbs) targeting uPAR block uPAR-mediated signaling, thereby inhibiting tumor cell migration and invasion. **(b)** Antibody-drug conjugates (ADCs) selectively deliver cytotoxic payloads to uPAR-expressing tumor cells, enabling targeted cytotoxicity. **(c)** Antibody-recruiting molecules (ARMs) redirect endogenous antibodies toward uPAR-expressing cells, promoting antibody-dependent cellular cytotoxicity (ADCC) and antibody-dependent cellular phagocytosis (ADCP). **(d)** uPAR-specific chimeric antigen receptor (CAR)-T cells recognize and eliminate both malignant and senescent uPAR-positive cells. The therapeutic rationale is based on three key observations: (1) elevated uPAR expression is associated with tumor invasion, metastasis, therapy resistance, and poor clinical outcome; (2) uPAR is expressed by both tumor cells and stromal components of TME, enabling dual targeting; and (3) uPAR-positive senescent stromal cells contribute to tumor progression and immune suppression, providing an additional therapeutic target.

### 3.1 Monoclonal antibodies

Monoclonal antibodies represent one of the earliest and most extensively investigated strategies for targeting uPAR. Among the first human antibodies shown to exhibit highly selective antagonistic activity were 2G10, 2E9, and 3C6. Specifically, 2G10 and 2E9 inhibit the interaction between uPA and uPAR, whereas 3C6 blocks the binding of β1 integrin to uPAR (Duriseti et al., 2010). LeBeau et al. demonstrated that treatment with 2G10 at a dose of 30 mg/kg inhibited the growth of triple-negative breast cancer (TNBC), while ^177^Lu-labelled 2G10 completely eradicated tumors in orthotopic breast cancer models (LeBeau et al., 2013). Monoclonal antibodies R3 and R5 represent the first proof-of-concept for uPAR-targeted antibodies that inhibit the uPA/uPAR axis through competitive and non-competitive mechanisms, respectively (List et al., 1999). Although these antibodies have been instrumental in elucidating the molecular mechanisms underlying uPA/uPAR signaling, they have remained primarily research tools rather than therapeutic candidates. More recent efforts have shifted toward the development of next-generation anti-uPAR antibodies, including humanized antibodies, which have demonstrated promising preclinical antitumor activity in several malignancies.

ATN-658, a mouse-derived IgG monoclonal antibody, binds with high affinity (*K*_d_ ∼ 1 nM) to DII–DIII of uPAR (Bauer et al., 2005). Unlike antibodies that directly compete with uPA, ATN-658 does not inhibit the uPA/uPAR interaction and is able to bind uPAR even when uPA is already bound. Despite this non-competitive binding mechanism, ATN-658 exhibited significant antitumor activity against human uPAR-expressing PC-3 (human prostate cancer cell line) xenograft tumors, demonstrating potent antagonistic effects *in vivo* (Rabbani et al., 2010). Subsequent structural studies of the Fab fragment demonstrated that ATN-658 binds to the DIII domain of uPAR, close to the C-terminus of the receptor and has no overlap with uPA and vitronectin binding sites (Xu et al., 2014). This study also revealed that the complementarity-determining region (CDR) loops of ATN-658 share homology with the uPAR-binding region of integrin CD11b (αM) (Xu et al., 2014). This suggests that ATN-658 binds to uPAR in a way that blocks or competes with the interaction between uPAR and integrin CD11b, thereby inhibiting the formation of the uPAR-integrin complex. Interestingly, ATN-658 was subsequently humanized to generate MNPR-101, which is currently in late-stage preclinical development. In preclinical models, MNPR-101 significantly inhibited the growth of MDA-MB-231 breast tumors. Furthermore, when combined with the bisphosphonate Zoledronic acid (Zometa), it markedly reduced breast cancer-induced bone lesions by suppressing osteoclast activity (Mahmood et al., 2020).

More recently, two potent fully human variable heavy (V_H_) domain antibodies targeting human uPAR were identified. Owing to their small size, high binding affinity, favorable expression efficiency, and low immunogenicity, V_H_ domains have emerged as promising scaffolds for antibody-based cancer immunotherapy. These antibodies were subsequently engineered into domain-based bispecific T-cell engagers (DbTEs), which mediated potent and selective T-cell cytotoxicity against tumor cells expressing detectable levels of uPAR. These findings highlight the potential of V_H_-based bispecific antibodies as a novel immunotherapeutic strategy for uPAR-positive malignancies (Chu et al., 2023).

### 3.2 Antibody-drug conjugates

Antibody-drug conjugates (ADCs), which deliver a targeted cytotoxic insult via drug-loaded tumor-homing monoclonal antibodies, have demonstrated substantial therapeutic efficacy in multiple solid malignancies (Fu et al., 2022). Nevertheless, despite considerable investigative efforts, only a limited number of tumor-associated surface antigens have been explored as ADC targets in solid tumors.

FL1-PNU is a site-specific ADC composed of the high-affinity anti-uPAR monoclonal antibody FL1 conjugated to a potent anthracycline derivative, enabling efficient target binding and rapid cellular internalization (Metrangolo et al., 2025). This ADC strategy demonstrated potent and specific antitumor activity in *in vitro* and *in vivo* models of pancreatic ductal adenocarcinoma (PDAC). Interestingly, PDAC exhibits the highest uPAR mRNA expression among all cancer types, with markedly elevated levels compared with normal pancreatic tissue and chronic pancreatitis. In addition, unlike established therapeutic targets such as HER2, uPAR expression is not restricted to malignant cells, as it is also highly expressed by several cellular components of the TME, including CAFs, macrophages, neutrophils, and endothelial cells (Metrangolo et al., 2021). Targeting uPAR with FL1-PNU in an orthotopic PDAC model resulted in marked tumor regression accompanied by extensive remodeling of the TME and significant immunomodulatory effects, characterized by increased infiltration of lymphocytes and pro-inflammatory immune cells, together with a reduction in immunosuppressive myeloid cell populations (Metrangolo et al., 2025).

Different ADCs have been developed by site-specific conjugation of the fully human anti-uPAR monoclonal antibody 2G10 to the microtubule inhibitor monomethyl auristatin E (MMAE) using distinct linker chemistries and conjugation strategies. Among these constructs, 2G10-RED-244-MMAE demonstrated the greatest antitumor activity, inducing marked tumor regression in preclinical models. The fully human nature of the 2G10 antibody minimizes the risk of immunogenicity, supporting its further development as a therapeutic candidate for aggressive malignancies, particularly triple-negative breast cancer (TNBC) (Harel et al., 2019).

To expand the repertoire of uPAR-targeting antibodies, Lourenço et al. employed high-throughput optofluidic screening of single B cells, a microfluidics-based technology that combines optical imaging with functional screening to rapidly identify and isolate rare antigen-specific antibody-producing B cells, leading to the identification of a panel of fully human, cross-reactive anti-uPAR monoclonal antibodies. The resulting candidates exhibited high-affinity binding, inhibition of uPA-uPAR interactions, antibody-dependent cellular cytotoxicity (ADCC), and favourable properties for ADC development. In a secondary ADC screening platform using an anti-human Fc Fab antibody conjugated to MMAE through a cathepsin-cleavable linker, three lead antibodies (3159, 8163, and 11857) displayed potent, concentration-dependent cytotoxicity against TNBC cells. Furthermore, these antibodies significantly suppressed tumor growth in an orthotopic human breast cancer model, highlighting their potential as next-generation platforms for the development of uPAR-targeted ADCs (Lourenço et al., 2023).

### 3.3 Antibody-recruiting molecules

Antibody-recruiting molecules (ARMs) represent an emerging immunotherapeutic strategy that redirects endogenous antibodies toward tumor cells, thereby promoting immune-mediated tumor elimination without the need for therapeutic monoclonal antibodies. Unlike antibody-drug conjugates, which rely on the intracellular delivery of cytotoxic payloads, ARMs exploit pre-existing circulating antibodies to trigger effector mechanisms such as ADCC and antibody-dependent cellular phagocytosis (ADCP) (Achilli et al., 2021).

Jakobsche and colleagues developed the first uPAR-targeting ARM, designated ARM-U1, by conjugating the amino-terminal fragment (ATF) uPA, which binds uPAR with high affinity, to the hapten 2,4-dinitrophenyl (DNP), a well-established antibody-recruiting moiety. By redirecting endogenous anti-DNP antibodies to uPAR-expressing tumor cells, ARM-U1 effectively induced ADCC and ADCP, thereby providing the first proof-of-concept for uPAR-targeted ARM technology (Jakobsche et al., 2012).

To overcome the limitations associated with the protein-based targeting moiety, the same group subsequently developed a second-generation construct, ARM-U2, in which the ATF domain was replaced by the high-affinity small-molecule uPAR ligand IPR-803. This design preserved efficient recruitment of endogenous antibodies while improving the pharmacological properties of the molecule, including its synthetic accessibility and potential for *in vivo* application. In a B16 melanoma allograft model engineered to express human uPAR, ARM-U2 achieved approximately 90% inhibition of tumor growth, highlighting the therapeutic potential of small-molecule antibody-recruiting strategies targeting uPAR (Rullo et al., 2016).

Collectively, these studies demonstrate that ARMs constitute a promising immunotherapeutic platform for uPAR-positive malignancies by combining inhibition of the uPA/uPAR signaling axis with immune-mediated destruction of tumor cells. Although this approach remains at the preclinical stage, its ability to harness endogenous humoral immunity without requiring engineered antibodies makes it an attractive and potentially cost-effective alternative for future cancer immunotherapy.

### 3.4 CAR-T cell therapy

Chimeric antigen receptor (CAR)-T cell therapy is a form of adoptive cellular immunotherapy in which autologous or allogeneic T lymphocytes are genetically engineered to express a synthetic receptor that recognizes predefined cell-surface antigens independently of major histocompatibility complex (MHC) presentation (Feins et al., 2019). A typical CAR comprises an extracellular antigen-binding domain, usually derived from a single-chain variable fragment (scFv) of a monoclonal antibody, linked through a hinge and transmembrane region to one or more intracellular signaling domains. This modular architecture enables engineered T cells to specifically recognize target antigens and initiate cytotoxic responses, cytokine secretion, and clonal expansion upon antigen engagement (Sadelain et al., 2013).

First-generation CARs contained only the CD3ζ signaling domain and demonstrated limited *in vivo* expansion and persistence (Brocker and Karjalainen, 1995). Second-generation CARs incorporated a single co-stimulatory domain, most commonly CD28 or 4-1BB (CD137), markedly improving T-cell proliferation, survival, and antitumor activity. These constructs form the basis of all currently approved CAR-T cell therapies (Maher et al., 2002). Third-generation CARs combine two co-stimulatory domains, such as CD28 and 4-1BB or CD28 and OX40 (CD134), with the aim of further enhancing T-cell activation, persistence, and cytokine production (Sterner and Sterner, 2021). More recently, fourth-generation CARs, also known as TRUCKs (T cells Redirected for Universal Cytokine-mediated Killing), have been engineered to express inducible cytokines or other immunomodulatory molecules following antigen recognition, thereby promoting immune cell recruitment and remodeling of the tumor microenvironment (TME) (Morgan and Schambach, 2018).

Among the various immunotherapeutic strategies targeting uPAR, CAR-T cell therapy has emerged as one of the most promising and rapidly evolving approaches. Although CAR-T cells have revolutionized the treatment of hematological malignancies, their clinical efficacy in solid tumors remains constrained by several factors, including antigen heterogeneity, an immunosuppressive TME, inadequate trafficking and tumor infiltration, and limited T-cell persistence (Srour and Akin, 2022). In this context, uPAR represents an attractive therapeutic target because of its broad expression not only on malignant cells but also on multiple stromal and immunosuppressive components of TME, includingCAFs, endothelial cells, TAMs, and senescent cells. This distinctive expression pattern offers the opportunity to simultaneously target tumor cells while disrupting the supportive tumor stroma, thereby potentially overcoming several of the major barriers that have thus far limited the efficacy of CAR-T cell therapy in solid tumors.

The first evidence supporting uPAR as a target for CAR-T cell therapy was provided by Wang et al., who investigated this approach in ovarian cancer. The authors developed a third-generation anti-uPAR CAR (ATF-CAR) in which the conventional scFv was replaced by ATF of uPA. ATF-CAR-T cells displayed potent antigen-specific and dose-dependent cytotoxicity against uPAR-positive ovarian cancer cells, demonstrating the feasibility of targeting uPAR through CAR-T cell therapy. The use of ATF, rather than an antibody-derived scFv, was intended to exploit the physiological uPA-uPAR binding interface, thereby potentially reducing immunogenicity. In addition, because ATF competitively binds the same receptor site as full-length uPA, this design may simultaneously interfere with uPA/uPAR signaling, providing the dual advantage of direct tumor cell killing and inhibition of pro-tumorigenic uPAR-mediated pathways (Wang L. et al., 2019).

Building on the initial demonstration that uPAR could serve as a target for CAR-T cell therapy in cancer, Amor et al. further expanded the therapeutic potential of this approach by exploiting uPAR as a marker of cellular senescence (Amor et al., 2024). The authors identified uPAR as a cell-surface protein consistently upregulated in senescent cells and developed a second-generation anti-uPAR CAR-T construct capable of selectively eliminating these cells. In preclinical models, uPAR-directed CAR-T cells efficiently cleared senescent cells, resulting in prolonged survival in mice with lung adenocarcinoma receiving senescence-inducing combination therapy and restoring tissue homeostasis in both chemically induced and diet-induced models of liver fibrosis. These findings established the first proof-of-concept that CAR-T cells can be engineered as senolytic agents. Moreover, given previous evidence that combining senescence-inducing anticancer therapies with senolytic agents improves therapeutic outcomes in preclinical models (Wang C. et al., 2019), this study provides a strong rationale for integrating senolytic uPAR-targeted CAR-T cells with conventional anticancer treatments to enhance the efficacy of solid tumor therapy.

Following the initial proof-of-concept studies, Maich et al. extended the application of uPAR-targeted CAR-T cells to recurrent glioblastoma (rGBM), a tumor characterized by profound intratumoral heterogeneity and a highly immunosuppressive microenvironment (Maich et al., 2026). To generate the CAR construct, the authors developed high-affinity camelid single-domain antibodies (sdAbs) against uPAR and incorporated them into a second-generation CAR backbone. Compared with conventional scFvs, sdAbs offer improved stability, solubility, and modularity while enabling recognition of unique antigenic epitopes. uPAR CAR-T cells efficiently recognized and eliminated patient-derived glioblastoma brain tumor-initiating cells (BTICs) both *in vitro* and in orthotopic xenograft models, resulting in significant tumor regression and prolonged survival following a single infusion. Importantly, the study also demonstrated that uPAR is highly expressed by TAMs-, particularly immunosuppressive M2-like macrophages, suggesting that uPAR-directed CAR-T cells can simultaneously target malignant cells and key cellular components of the glioblastoma microenvironment (Maich et al., 2026).

In the study by Zhang et al., uPAR-directed CAR-T cells were engineered using a second-generation CAR platform designed to target the D2-D3 domains of uPAR (Zhang Z. et al., 2026). uPAR CAR-T cells exhibited broad antitumor activity across multiple solid tumor models, including xenograft, syngeneic, and patient-derived systems. Importantly, the therapeutic efficacy of this approach was attributed not only to the elimination of uPAR-expressing tumor cells but also to the targeting of uPAR-positive stromal populations. Furthermore, uPAR CAR-T cells induced durable tumor regressions, an effect that was further enhanced by senescence-inducing therapies.

More recently, the therapeutic potential of uPAR-targeted CAR-T cells has been extended to the field of healthy aging. In a recent study, the authors developed a senolytic CAR-T platform by engineering human T cells to express a second-generation anti-mouse uPAR CAR comprising an anti-uPAR single-chain variable fragment (scFv) fused to the human CD28 co-stimulatory and CD3ζ signaling domains. The resulting CAR-T cells selectively and efficiently eliminated uPAR-positive senescent cells both *in vitro* and *in vivo*. Using this approach, the authors demonstrated that uPAR-positive epithelial cells accumulate in the aging intestines of both mice and humans, where they acquire a senescence-like phenotype that contributes to chronic inflammation, epithelial dysfunction, and impaired tissue regeneration. Selective elimination of these cells restored intestinal stem cell function, improved epithelial regeneration and barrier integrity, reduced age-associated inflammation and dysbiosis, and ultimately enhanced tissue fitness. These findings support the concept that uPAR-directed CAR-T cells may represent a promising senolytic strategy to promote healthy aging and extend healthspan (Eskiocak et al., 2026).

Despite these encouraging preclinical results, several limitations and safety concerns should be considered. First, although uPAR expression is relatively enriched in tumor tissues compared with most normal tissues, it is not completely tumor-specific. uPAR is expressed at low levels in certain normal cell populations, particularly myeloid cells, and is also upregulated in senescent cells that may accumulate in normal tissues during aging or tissue injury. Therefore, uPAR-directed CAR-T cells may carry a potential on-target/off-tumor risk, particularly in settings characterized by increased senescence or inflammation. Recent studies have nevertheless reported limited toxicity and preserved tissue integrity following uPAR CAR-T administration in preclinical models, suggesting a potentially favorable therapeutic window; however, these findings require careful validation in humans (Hu et al., 2014; Zeng et al., 2021). In addition, the expression of uPAR on immunosuppressive myeloid populations may represent a double-edged sword: their elimination could contribute to remodeling of TME, but systemic targeting of uPAR-positive myeloid cells could also affect physiological immune functions.

A second major challenge is the limited trafficking and penetration of CAR-T cells into solid tumors. Dense ECM, stromal barriers and immunosuppressive cellular components can restrict CAR-T-cell infiltration and impair their persistence and effector function within TME. Although uPAR-directed CAR-T cells can target both malignant cells and uPAR-positive stromal populations, this broad targeting capacity does not necessarily overcome the physical barriers that limit T-cell access to poorly vascularized or highly fibrotic tumor regions. Notably, recent work suggests that combining uPAR CAR-T cells with senescence-inducing therapies can increase uPAR expression and improve CAR-T-cell infiltration, providing a potential strategy to enhance both target accessibility and intratumoral activity (Zhang Z. et al., 2026).

Third, antigen heterogeneity and antigen escape remain important potential mechanisms of resistance. Although uPAR is expressed across multiple tumor and stromal compartments, its expression is heterogeneous within and between tumors, and cells with low or absent uPAR expression may escape CAR-T-cell-mediated elimination. Moreover, selective pressure exerted by CAR-T cells could favor the expansion of uPAR-low or uPAR-negative tumor cell populations. Targeting uPAR-positive stromal cells in addition to tumor cells may reduce this risk by simultaneously disrupting the tumor-supportive niche; however, it cannot completely prevent the emergence of antigen-low tumor clones. Strategies aimed at increasing uPAR expression, such as senescence-inducing therapies, or combining uPAR targeting with CARs directed against additional tumor antigens may therefore be required to limit antigen escape and improve the durability of responses (Zhang Z. et al., 2026).

Finally, the choice of extracellular antigen-recognition domain is an important determinant of CAR-T cell performance, as each recognition modality offers distinct advantages and limitations. The ATF-based approach exploits the natural uPA-uPAR binding interface and may provide favorable target specificity while potentially reducing immunogenicity compared with non-human antibody-derived binders. In addition, because ATF competes with endogenous uPA for uPAR binding, it may provide the additional benefit of interfering with uPA/uPAR-mediated pro-tumorigenic signaling. However, the use of a natural ligand may also affect uPAR biology and requires careful evaluation of its pharmacological and safety properties (Wang L. et al., 2019). sdAbs offer a substantially smaller and structurally compact antigen-binding format, with favorable stability, solubility, and the potential to access epitopes that may be less accessible to conventional antibody fragments. Nevertheless, the potential contribution of conserved framework residues to binding affinity without necessarily conferring antigen specificity raises important questions regarding the molecular basis of sdAb specificity, with potential implications for the design and engineering of sdAb-based therapeutics (Gordon et al., 2023). scFvs represent the most widely used and readily engineerable CAR recognition modality due to several advantages such as a high specificity and affinity for antigens, a low immunogenicity, and the proven ability to penetrate tumor tissues and diffuse (Muñoz-López et al., 2022). However, scFv performance can be influenced by linker composition, which may affect folding, stability, and solubility, as well as by the absence of the Fc domain, which can complicate purification. Furthermore, scFvs may be prone to aggregation and misfolding, potentially compromising their stability and increasing the risk of immunogenicity (Gezehagn Kussia and Tessema, 2024). Moreover, very high-affinity binding does not necessarily translate into optimal tumor penetration or therapeutic efficacy. Thus, the selection of the optimal uPAR recognition domain should balance affinity and specificity with stability, immunogenicity, epitope accessibility, tumor penetration, and safety.

### 3.5 Combination strategies and emerging immunotherapeutic platforms

Despite the encouraging preclinical activity of uPAR-targeted immunotherapies, their efficacy as monotherapies may be limited by tumor heterogeneity, antigen escape, and the highly immunosuppressive TME. Therefore, increasing attention has been directed toward combination strategies and next-generation immunotherapeutic platforms aimed at enhancing antitumor activity, improving immune cell recruitment and persistence, and overcoming mechanisms of therapeutic resistance.

Among combination approaches, the integration of uPAR blockade with immune checkpoint inhibition represents one of the most promising strategies. A monoclonal antibody targeting DII-DIII of uPAR, combined with anti-PD-1 therapy, significantly inhibited tumor growth and prolonged survival in both cell line-derived xenograft (CDX) and patient-derived xenograft (PDX) models of diffuse-type gastric cancer. Interestingly, anti-uPAR treatment alone or in combination with PD-1 blockade increased the infiltration of CD8^+^ TILs, with the combination regimen producing an additive effect. These findings indicate that uPAR targeting may not only interfere with tumor-promoting pathways but also remodel the immune landscape of TME, thereby enhancing T-cell infiltration and providing a strong rationale for combining uPAR-directed therapies with immune checkpoint inhibitors (Qin et al., 2022).

In parallel with antibody-based approaches, innovative nucleic acid-based immunotherapeutic platforms are expanding the therapeutic potential of uPAR targeting. A notable example is the development of a bifunctional 5′-triphosphate-conjugated small interfering RNA (ppp-uPAR), which simultaneously suppresses PLAUR expression and activates the cytosolic pattern-recognition receptor retinoic acid-inducible gene I (RIG-I). This dual mechanism combines inhibition of uPAR-mediated oncogenic signaling with innate immune activation, resulting in enhanced type I interferon responses and apoptosis induction in melanoma cells. Importantly, ppp-uPAR retained antitumor activity in melanoma models resistant to BRAF and MEK inhibitors and significantly reduced tumor growth *in vivo* while inducing systemic innate immune responses (Matheis et al., 2016).

Beyond conventional immune activation strategies, emerging nanotechnology-based platforms provide new opportunities to enhance the precision and efficacy of uPAR-directed immunotherapies. Rather than representing immunotherapies themselves, uPAR-targeted nanoplatforms can serve as enabling technologies for selective delivery of immunomodulatory cargos and for remodeling of tumor-associated cellular states. A recent study described a uPAR-targeted nanotherapeutic strategy that selectively recognized uPAR-expressing senescent tumor cells and delivered gene-modulating cargos capable of reversing senescence-associated immunosuppressive programs. By reprogramming the phenotype of these senescent tumor cells, the treatment enhanced immune surveillance and promoted immune-mediated tumor control, highlighting the potential of uPAR-targeted nanoplatforms as tools for tumor microenvironment reprogramming (Zhao et al., 2026).

Furthermore, advances in nanoparticle-mediated immune cell engineering have opened new perspectives for uPAR-directed cellular therapies. PL40 lipid nanoparticles were developed to deliver mRNA encoding a uPAR-specific CAR, enabling the *in vivo* generation of CAR-T cells without the need for *ex vivo* manufacturing. This platform exploited uPAR as a marker of senescent and inflammatory cell populations and demonstrated therapeutic efficacy in preclinical models of uPAR-associated inflammatory disorders, including liver fibrosis and rheumatoid arthritis. Single-cell transcriptomic analyses further supported the involvement of uPAR in senescence-associated inflammatory programs, while the development of humanized anti-uPAR single-chain variable fragments (scFvs) facilitated the translational potential of this platform (Zhang et al., 2025). Although primarily investigated in non-oncological settings, this strategy provides a conceptual framework for the future development of *in vivo* uPAR-directed CAR-T therapies for solid tumors.

Similarly, uPAR-targeted hybrid nanoparticles co-encapsulating the senolytic agent dasatinib and quercetin have been developed to selectively eliminate uPAR-expressing senescent cells. This targeted delivery strategy reduced inflammatory signaling and restored metabolic homeostasis in preclinical models of aging- and obesity-associated disorders, highlighting the potential of exploiting uPAR as a molecular address for senolytic therapy (Jung et al., 2026). In the oncology setting, where senescent tumor and stromal cells contribute to chronic inflammation, immune dysfunction, and therapeutic resistance, such approaches may offer a complementary strategy to enhance immune surveillance, remodel the tumor microenvironment, and improve responses to immune checkpoint blockade or adoptive cell therapies.

The high expression of uPAR has been exploited as a targeting receptor for the selective delivery of chemotherapy in pancreatic ductal adenocarcinoma. A pH-responsive, uPAR-targeted polymeric micellar system encapsulating gemcitabine (Gem@TpHResMic) showed enhanced cellular uptake, controlled drug release in the acidic tumor microenvironment, and superior antitumor activity compared with free gemcitabine in both 2D and 3D models of pancreatic ductal adenocarcinoma. Importantly, the targeted nanocarrier was able to overcome CAF-mediated drug resistance, highlighting uPAR as an attractive target not only for active drug delivery but also for improving therapeutic efficacy within the highly desmoplastic pancreatic ductal adenocarcinoma microenvironment (Iacobazzi et al., 2021). Similarly, a pH-sensitive liposomal formulation encapsulating cisplatin (Pt), designated ATF@Pt Lps, was developed to selectively target uPAR-expressing pancreatic cancer cells, TAMs, and CAFs. ATF@Pt Lps effectively penetrated the dense stromal barrier, facilitating enhanced drug delivery to tumor cells and producing a robust therapeutic effect in a cell-derived orthotopic mouse model of pancreatic cancer. Notably, ATF@Pt Lps promoted collagen degradation within TME, thereby enhancing the infiltration of CD8^+^ T cells and increasing the intratumoral accumulation of anti-PD-1 monoclonal antibodies. In addition, cisplatin-induced tumor cell death triggered the release of tumor-associated antigens, stimulating antitumor immune responses characterized by the expansion of immune effector populations, including CD83^+^ dendritic cells, Th1-polarized CD4^+^ T cells, and CD8^+^ cytotoxic T lymphocytes, thereby improving responsiveness to immunotherapy (Yu et al., 2023).

Finally, uPAR-targeted vaccines represent another emerging strategy aimed at inducing adaptive immune responses against uPAR-expressing malignant cells. A multiepitope vaccine targeting uPAR has been proposed by integrating predicted T-cell epitopes with a TLR4 agonist as an immunostimulatory adjuvant. Computational immune simulations suggested that this vaccine could generate sustained antibody responses, activate cellular immunity, and promote long-term immunological memory, supporting its potential as a candidate strategy for uPAR-directed cancer immunotherapy (Baghaei et al., 2025). However, experimental validation in appropriate *in vitro* and *in vivo* tumor models will be required to confirm its immunogenicity, safety profile, and therapeutic efficacy.

Collectively, these findings illustrate the expanding landscape of uPAR-directed immunotherapy, in which combination regimens, biomarker-guided patient selection, innate immune activation, vaccine-based approaches, cellular engineering, and nanotechnology-enabled platforms may converge to overcome current limitations of immunotherapy in solid tumors.

## 4 uPAR as biomarker in the context of immunotherapy

Beyond its role as a therapeutic target, uPAR has emerged as a promising biomarker in cancer immunotherapy because it can provide clinically relevant information through both circulating and imaging-based approaches. As a multifunctional receptor involved in tumor progression, ECM remodeling, and immune regulation, uPAR reflects not only the intrinsic biological characteristics of tumor cells but also the dynamic interactions within TME. Its translational value lies in the availability of complementary biomarker platforms. Membrane-bound uPAR can be non-invasively visualized using targeted molecular imaging, enabling whole-body assessment of receptor expression, disease burden, and intratumoral heterogeneity (Baart et al., 2020). Conversely, proteolytic cleavage of the receptor generates soluble uPAR (suPAR), which can be readily quantified in plasma or serum as a minimally invasive indicator of systemic immune activation and tumor-associated inflammation (Rasmussen et al., 2021).

Supporting the clinical relevance of uPAR as a biomarker, a recent radiogenomic study in intrahepatic cholangiocarcinoma identified *PLAUR* as a key molecular determinant associated with response to immunochemotherapy. Integrative analyses combining radiological features with transcriptomic profiling demonstrated that elevated *PLAUR* expression was associated with a distinct immune landscape and increased therapeutic responsiveness, highlighting uPAR as both a potential therapeutic target and a biomarker for patient stratification (Ji et al., 2025). Additional evidence has been provided by studies investigating extracellular vesicles (EVs). In patients with metastatic melanoma receiving immune checkpoint inhibitors, elevated levels of uPAR-positive EVs were associated with primary resistance and inferior clinical outcomes, suggesting that these vesicles may serve as a liquid biopsy biomarker for predicting response to checkpoint blockade and supporting therapeutic decision-making (Porcelli et al., 2021).

Among circulating biomarkers, suPAR has emerged as a promising candidate in the context of cancer immunotherapy. Elevated baseline plasma suPAR concentrations have been consistently associated with inferior clinical outcomes in patients with advanced solid tumors receiving immune checkpoint inhibitors. Conversely, patients with low baseline or on-treatment suPAR levels exhibited significantly prolonged progression-free survival (PFS) and overall survival (OS), suggesting that circulating suPAR reflects biological processes associated with resistance to immunotherapy. These findings support the role of suPAR as a readily accessible prognostic biomarker for risk stratification and clinical outcome prediction in treated patients. However, the available evidence predominantly supports its prognostic rather than predictive value, and whether suPAR independently predicts therapeutic response to immune checkpoint blockade remains to be determined in prospective clinical studies (Loosen et al., 2021).

suPAR has also emerged as a potential biomarker in adoptive cellular immunotherapy. In recipients of CAR-T cell therapy, suPAR concentrations were significantly increased and strongly correlated with established indicators of endothelial dysfunction, including the modified Endothelial Activation and Stress Index (mEASIX) and EASIX scores. These observations indicate that suPAR reflects CAR-T cell-associated endothelial activation and vascular injury and may therefore represent a valuable biomarker for monitoring treatment-related endothelial toxicity and identifying patients at increased risk of severe complications (Gavriilaki et al., 2024).

In parallel, uPAR-targeted molecular imaging is emerging as a promising companion biomarker for cancer immunotherapy, providing a non-invasive approach to characterize target expression and tumor biology at the whole-body level. By enabling quantitative visualization of uPAR expression across primary and metastatic lesions, molecular imaging captures both spatial and temporal heterogeneity while monitoring dynamic changes within TME, thereby overcoming the sampling limitations inherent to conventional tissue biopsy. This capability may improve patient selection, enable longitudinal assessment of pharmacodynamic responses and treatment-induced biological remodeling, and support response prediction, therapeutic monitoring, and the implementation of adaptive precision immunotherapy strategies (Camedda et al., 2024).

The clinical translation of uPAR-targeted molecular imaging has progressed rapidly over the past decade. Persson et al. conducted the first-in-human phase I clinical trial of the uPAR-targeted radioligand ^64^Cu-DOTA-AE105 in 10 patients with solid tumors, evaluating its safety, pharmacokinetics, dosimetry, and tumor uptake (Persson et al., 2015). The radiotracer showed favorable biodistribution, high tumor-to-background ratios, and enabled visualization of both primary tumors and metastatic lymph nodes, with no treatment-related adverse events reported. However, the limited availability of ^64^Cu prompted the development of alternative radiolabeling strategies, leading to subsequently introduce the ^68^Ga-labeled uPAR ligand. In 2017, the first-in-human phase I clinical trial of ^68^Ga-NOTA-AE105 demonstrated its safety, feasibility, and clinical potential for uPAR PET imaging in patients with solid tumors (Skovgaard et al., 2017). Subsequent clinical studies further highlighted its prognostic value. In patients with head and neck squamous cell carcinoma, ^68^Ga-uPAR PET SUV_max_ outperformed conventional prognostic parameters in predicting recurrence-free survival (RFS), underscoring the potential of uPAR-targeted imaging for patient risk stratification and treatment personalization (Risør et al., 2022). The radiolabeled compounds developed to date have demonstrated a favorable safety profile, acceptable dosimetry, and effective tumor detection. Notably, ^68^Ga-NOTA-AE105 offers several advantages over its ^64^Cu-labeled counterpart, including lower cost, greater availability, and more favorable biodistribution. Its minimal hepatobiliary and gastrointestinal excretion may facilitate the identification of pathological tracer uptake in the abdomen and pelvis. However, the sensitivity of uPAR-targeted radiotracers may vary across tumor types, as ^68^Ga-uPAR PET showed limited efficacy in detecting liver metastases from disseminated bladder cancer (Skovgaard et al., 2017). Further research is therefore needed to address the current limitations, including the relatively limited number of clinically validated agents, differences in pharmacokinetics and tumor-to-background contrast, and the small patient cohorts, as only two studies included more than 50 patients. Moreover, all available clinical studies were conducted in Denmark by the same research group, highlighting the need for independent and multicenter validation (Camedda et al., 2024).

More recently, the translational applications of uPAR imaging have been extended to the detection of therapy-induced senescence. Using an anti-uPAR immuno-PET approach, investigators selectively visualized senescent pancreatic cancer cells following treatment with trametinib and palbociclib, demonstrating therapy-induced upregulation of uPAR *in vivo* (Pratt et al., 2024). Although the study was not conducted in the context of immunotherapy, it provides proof-of-concept for the use of uPAR immuno-PET as a companion imaging biomarker for immune-based therapeutic strategies. Given the contribution of senescent uPAR-positive cells to tumor persistence, immune remodeling, and therapeutic resistance, non-invasive visualization of these cell populations could improve patient stratification, identify residual treatment-resistant lesions, and enable longitudinal monitoring of emerging uPAR-targeted immunotherapies, including senolytic CAR-T cell approaches.

When considered together, suPAR, uPAR-targeted PET imaging, and uPAR-positive EVs offer complementary advantages as uPAR-based biomarkers, but differ substantially in their degree of invasiveness, technical requirements, clinical applicability, and predictive potential. Circulating suPAR can be measured from minimally invasive blood samples using relatively accessible and standardized immunoassays, making it the most readily applicable and potentially cost-effective approach for repeated measurements and longitudinal monitoring (Napolitano and Montuori, 2026). However, because suPAR is released by multiple cell types and reflects systemic inflammatory and immune states in addition to tumor biology, its specificity for tumor burden and treatment response may be limited. In contrast, uPAR-targeted PET provides non-invasive, whole-body visualization and quantitative assessment of uPAR expression, allowing spatial characterization of primary and metastatic lesions and potentially overcoming the sampling limitations of tissue biopsy. Nevertheless, PET imaging requires specialized infrastructure, radiotracer production and expertise, resulting in substantially greater technical complexity and cost than circulating biomarker assays. uPAR-positive EVs represent an intermediate and potentially complementary approach, as their detection in liquid biopsies combines minimally invasive sampling with the possibility of capturing membrane-associated uPAR and other tumor-derived molecular cargo within extracellular vesicles. However, EV isolation and characterization remain technically demanding, lack complete standardization across platforms, and may limit their current routine clinical implementation. From a clinical perspective, these approaches may therefore serve different purposes: suPAR may be particularly useful for systemic risk stratification and longitudinal monitoring, uPAR-targeted PET for non-invasive assessment of target distribution, tumor heterogeneity and treatment-induced changes, and uPAR-positive EVs for more specific molecular characterization of circulating tumor-derived material.

Collectively, these findings position uPAR as a versatile biomarker platform that integrates liquid biopsy and molecular imaging approaches, bridging molecular diagnostics with precision immunotherapy. By enabling patient stratification, treatment monitoring, and real-time assessment of target expression, uPAR-based biomarkers may facilitate the implementation of personalized immunotherapeutic strategies and accelerate the clinical translation of uPAR-directed therapies.

## 5 Concluding remarks

uPAR represents an attractive therapeutic target in oncology owing to its preferential expression in malignant tissues and its multifaceted role in tumor progression. Beyond regulating cell proliferation, survival, invasion, metastasis, and therapy resistance, uPAR orchestrates complex interactions between cancer cells and TME, providing a strong biological rationale for the development of diverse uPAR-directed therapeutic strategies. In recent years, substantial advances have been achieved in the fields of antibody-based therapies, antibody-drug conjugates, bispecific antibodies, CAR-T cells, and molecular imaging, underscoring the versatility of uPAR as both a therapeutic target and a biomarker. Although these approaches have demonstrated encouraging efficacy in preclinical models, none have yet entered clinical evaluation in oncology. Nevertheless, the recent humanization of the anti-uPAR monoclonal antibody ATN-658 (huATN-658) represents an important milestone toward clinical translation and provides a platform for the future development of uPAR-directed therapeutics (Xu et al., 2014).

Despite these advances, several biological and clinical challenges must be overcome before uPAR-targeted therapies can be successfully implemented in routine clinical practice. A major limitation is the pronounced intra- and intertumoral heterogeneity of uPAR expression, which may enable uPAR-low or uPAR-negative tumor cell populations to evade therapeutic pressure and promote disease recurrence (Zhai et al., 2022). In addition, the potential for on-target/off-tumor toxicity remains an important safety concern because low physiological uPAR expression is retained in several normal tissues, including endothelial cells, activated leukocytes, renal tubular epithelium, and liver sinusoidal cells (Zhai et al., 2022). Therapeutic efficacy is further constrained by the immunosuppressive TME, where ECM deposition, hypoxia, immunoregulatory cytokines, and suppressive immune cell populations collectively impair the trafficking, persistence, and effector function of uPAR-targeted immune cells (Johnson et al., 2022). Moreover, sustained therapeutic pressure may promote antigen escape through downregulation of uPAR expression, genetic adaptation, or increased shedding of soluble uPAR (suPAR).

Future clinical development will likely rely on combination strategies rather than monotherapy. Similar to immune checkpoint inhibitors, whose efficacy is frequently enhanced by rational combinations, uPAR-targeted therapies are expected to benefit from integration with chemotherapy, radiotherapy, targeted agents, or complementary immunotherapeutic approaches. Equally important will be the implementation of precision medicine strategies supported by robust biomarker platforms. Standardized immunohistochemistry, transcriptomic profiling, circulating suPAR measurements, extracellular vesicle analysis, and uPAR-targeted molecular imaging, including uPAR PET, may enable accurate patient stratification, longitudinal monitoring of target expression, and real-time assessment of therapeutic responses. The integration of these complementary biomarkers with next-generation uPAR-directed therapeutics has the potential to maximize clinical efficacy, minimize unnecessary toxicity, and accelerate the translation of uPAR-targeted immunotherapy into precision oncology.
